# Iatrogenic Radial Nerve Palsy following Closed Reduction of a Simple Diaphyseal Humeral Fracture: Beware the Perfect X-Ray

**DOI:** 10.1155/2016/2636450

**Published:** 2016-07-12

**Authors:** Morgan Jones, Hean Wu Kang, Christopher O'Neill, Paul Maginn

**Affiliations:** ^1^Fractures Department, Royal Victoria Hospital, Belfast BT12 6BA, UK; ^2^Ulster Hospital, Dundonald, Belfast BT16 1RH, UK

## Abstract

Radial nerve injury is a recognised complication associated with humeral shaft fracture. A case of iatrogenic radial nerve injury is presented following fracture reduction. The relevant anatomy, challenges in management of humeral fractures with associated radial nerve injury, and the importance of detailed clinical assessment and documentation are discussed.

## 1. Introduction

**Figure 1:**
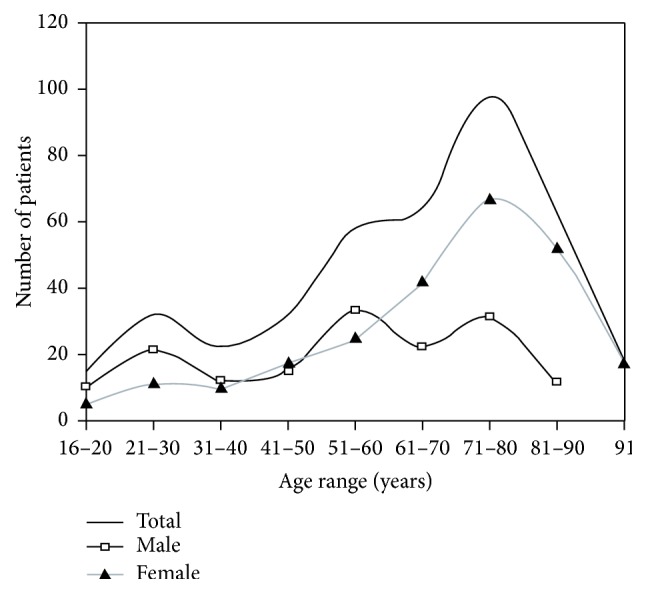
Age distribution of humeral shaft fractures.

Humeral shaft fractures account for 1–3% of all fractures [1]. The age distribution follows a bimodal pattern, with peaks in the younger patient population following high energy trauma and the elderly osteopenic patient population following low energy trauma (see Figure 1) [1]. Management is dependent upon patient age, hand dominance, occupation, fracture pattern, fracture displacement, and associated injuries. The majority of cases are low energy simple fractures that are suitable for conservative rather than operative management [2].

With appropriate management, the clinician can expect excellent outcomes and overall union rates of greater than 90% [2, 3]. Radial nerve injury is reported in up to 12% of cases of diaphyseal humeral fractures and is associated with significant morbidity and adverse functional outcomes [1, 4].

## 2. Case Presentation

A 61-year-old right hand dominant housewife sustained an injury to her right upper limb following a low energy mechanical fall.

**Figure 2:**
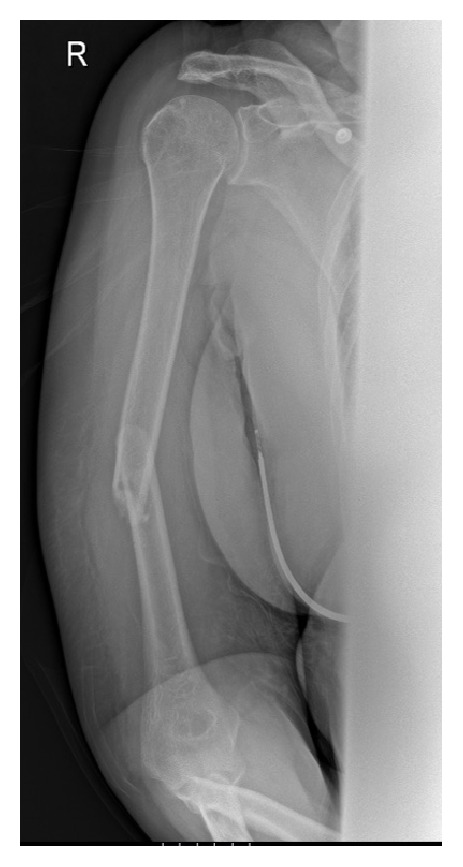
Initial AP radiograph showing a mid-diaphyseal humeral fracture with mild angulation.

The patient attended the Emergency Department (ED) due to significant right arm pain, swelling, and deformity. No neurovascular deficit was documented on initial clinical assessment. X-rays confirmed a short spiral fracture of the humeral diaphysis with slight angulation (Figure 2).

**Figure 3:**
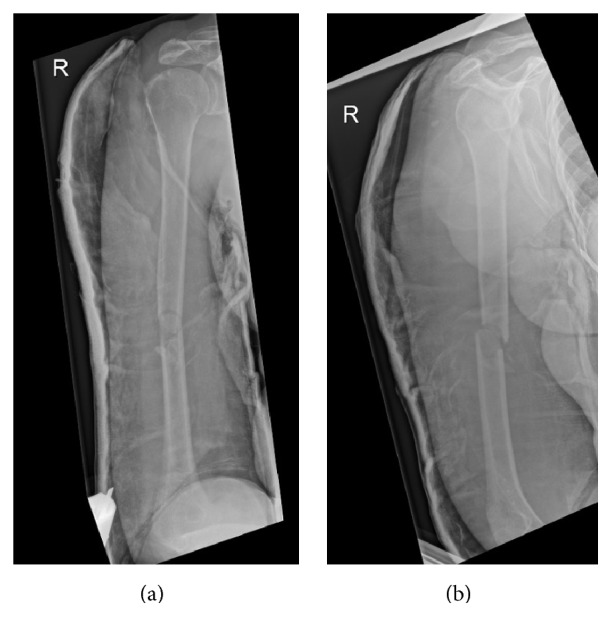
Post-reduction radiographs demonstrating excellent alignment.

A Bohler-U cast was applied and repeat X-ray demonstrated excellent alignment on both anteroposterior and lateral views (Figures 3(a) and 3(b)). On return from the X-ray department the patient reported new onset of paraesthesia in the right hand and difficulty moving her fingers. On detailed questioning, the patient admitted to very slight alteration of sensation at the thumb base and posterior aspect of the forearm prior to application of the Bohler-U cast. Examination confirmed MRC grade 0/5 power in wrist and finger extensors and reduced sensation at the base of thumb and posterior aspect of the forearm in keeping with a dense radial nerve palsy. Peripheral pulses were easily palpable and capillary refill was normal.

**Figure 4:**
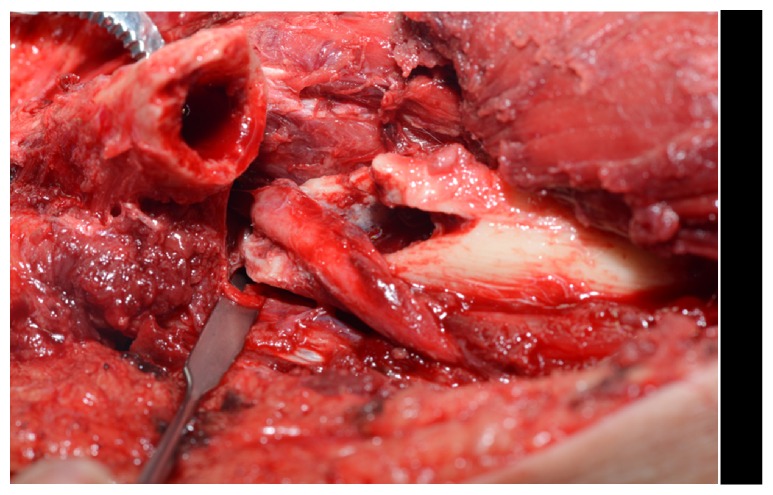
Intraoperative photograph demonstrating interposition of the radial nerve at the fracture site with radial nerve contusion.

Due to the onset of new neurologic deficit following fracture reduction and cast application, the clinical concern was of iatrogenic nerve injury. Urgent surgical exploration was performed via an anterolateral approach. Intraoperatively, the radial nerve was found to be interposed in the fracture (Figure 4).

**Figure 5:**
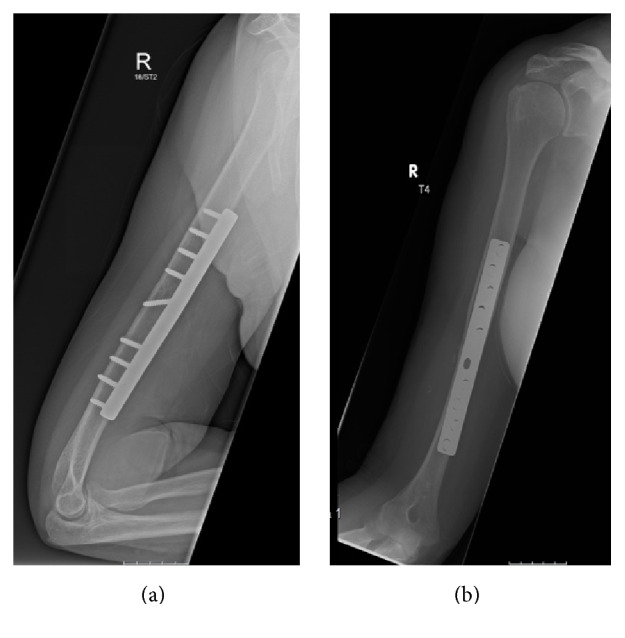
Radiographs demonstrating surgical fixation with a dynamic compression plate.

The nerve was in continuity, though there was evidence of significant contusion. Following careful extraction of the radial nerve, the fracture was reduced and stabilised using a 10-hole AO (Arbeitsgemeinschaft für Osteosynthesefragen) large fragment dynamic compression plate and interfragmentary compression screw (Figure 5).

Postoperatively, there was no immediate improvement in radial nerve function. A custom-made thermoplastic wrist extension splint was initially fitted. Examination at one week from date of surgery demonstrated some early return of sensation in the distribution of the superficial branch of the radial nerve though the dense motor deficit remained unchanged. An early rehabilitation programme consisting of passive range of movement exercises and dynamic splinting was commenced under the supervision of departmental hand therapists. Full active shoulder and elbow range of movement exercises commenced 4 weeks from date of surgery. Examination at 3 months demonstrated MRC grade 3/5 finger extension and wrist extension. At 3 months from date of surgery, the fracture also appeared radiologically united. Complete recovery of radial nerve function was evident at 8 months (MRC grade 5/5).  Table 1 highlights our patient's recovery process as described above.

## 3. Discussion

### 3.1. Neural Anatomy

There are three connective tissue layers in a central or peripheral nerve. On the simplest level, a single nerve axon together with its myelin sheath are covered by the* endoneurium*. Multiple nerve axons bundle together to form fascicles, which are covered by the* perineurium.* Finally, in sufficiently large nerves, groups of fascicles congregate and are protected by an* epineurium* sheath with its own blood supply and fatty tissue.

### 3.2. Nerve Injury Classification and Management

The Seddon classification describes three degrees of nerve injury: neurapraxia, axonotmesis, and neurotmesis.

Neurapraxia is a first-degree injury where nerve compression occurs, often due to blunt trauma to the nerve. Temporary conduction block occurs due to local demyelination; however axonal continuity remains intact. Sensory, more than motor, problems develop distal to site of injury. Because no Wallerian degeneration occurs, recovery is often complete with recovery times ranging from hours to weeks.

Axonotmesis is a second-degree injury where axonal and myelin continuity are lost, but the connective tissue layers (basement membrane) encapsulating the neural tubes remain intact. Both sensory and motor dysfunctions are present; however axonal regeneration occurs due to release of chemotactic and trophic growth factors from distal targets of peripheral nerves. Recovery can take weeks to months, and full recovery is often anticipated without surgical intervention, although it may be required if scar formation blocks the path of axonal regeneration. The patient in this case report suffered an axonotmesis.

Neurotmesis, a third-degree injury, is the most severe form. There is complete and physical detachment of the nerve and all its structures. Recovery, often incomplete and imperfect, and usually requires surgical intervention. Clinically, both axonotmesis and neurotmesis present similarly, with motor and sensory deficits, paralysis, and muscle atrophy.

### 3.3. Gross Anatomy

Radial nerve interposition at the site of a humeral shaft fracture is a rare but recognised complication. Holstein and Lewis first described a simple spiral fracture in the distal third of the humerus associated with radial nerve injury in 1963 [5]. More recent reviews have highlighted the risk of radial nerve injury following diaphyseal fractures [4].

The radial nerve arises from the posterior cord of the brachial plexus (C5-T1), lying behind the axillary artery. It courses on the posterior wall of the axilla running on subscapularis, latissimus dorsi, and teres major. It gives off three branches in the axilla: two to triceps and the posterior cutaneous nerve of the arm. Following this the nerve then runs through the triangular interval with the profunda brachii artery to enter the posterior compartment of the arm. At this point it is intimately associated with the humeral shaft coursing through the spiral groove between the lateral and medial head of triceps. The nerve then passes through the lateral intermuscular septum, approximately 8 cm proximal to the lateral epicondyle. Once passing through the intermuscular septum, the nerve runs between brachialis and brachioradialis where it gives off branches supplying lateral brachialis, brachioradialis, extensor carpi radialis longus, and extensor carpi radialis brevis. The nerve then passes anterior to the radiohumeral joint and divides into the posterior interosseous nerve and its terminal superficial sensory branch [6] (Figure 7).

Both the spiral groove and the point at which the radial nerve traverses the intermuscular septum are anatomic regions where the nerve is relatively fixed or tethered. Fractures in close proximity to these regions can make the nerve more susceptible to indirect injury as a result of traction at time of maximal deformity or direct injury due to laceration from bony spikes or fracture interposition.

### 3.4. Management Considerations

The majority of diaphyseal humeral fractures can me managed conservatively with Bohler-U/functional bracing [2]. Surgical fixation typically involves the use of either a plate or an intramedullary nail. Relative indications for surgical fixation are dependent upon age, occupation, and patient preference. Absolute indications for surgical intervention include open or vascular injury and brachial plexus injury [7, 8].

There is considerable debate regarding management of nerve injury that has occurred at the time of fracture. There are broadly speaking two schools of thought—early exploration of the radial nerve versus taking a delayed approach with surgical repair or tendon transfer at a later date dependent on residual nerve function.

With a radial nerve injury sustained at the time of injury, initial nonoperative or expectant treatment is advocated by the majority of surgeons as there is a high rate of spontaneous recovery of radial nerve function. With expectant treatment, a significant number of cases will therefore avoid the need surgical intervention and the potential complications associated with it. The literature also suggests that recovery of neurological function is equivalent with either early or late repair [4, 10].

A proportion of surgeons advocate early nerve exploration, believing that early exploration is technically easier and safer than a delayed procedure. Early exploration also allows direct visualisation of the nerve and further defines the extent of macroscopic injury. Operative fracture fixation reduces potential trauma to the nerve from mobile bone ends and minimises the risk of the nerve entrapped in scar tissue or fracture callus. Acute humeral shortening to facilitate nerve repair is also preferable prior to fracture healing [4].

**Figure 6:**
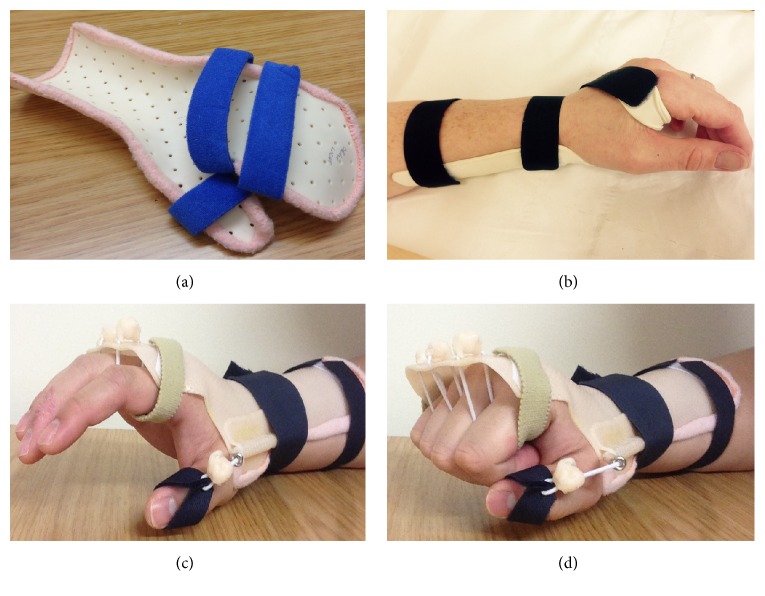
Examples of night, resting, and dynamic orthoses used in patients with a radial nerve deficit.

**Figure 7:**
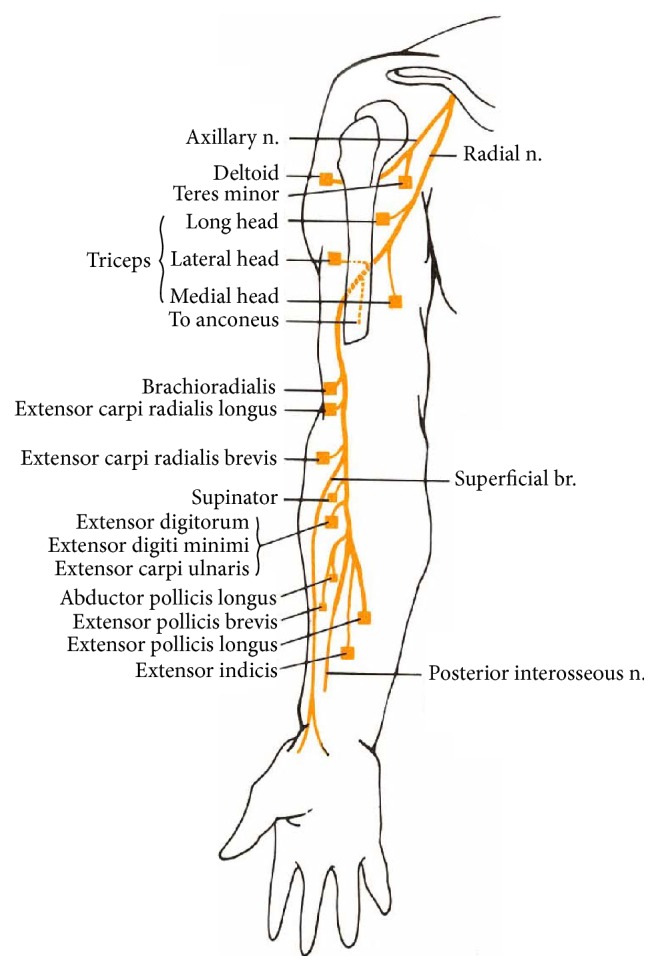
Journey of the radial nerve from the posterior cord of the brachial plexus to the terminal branches in the forearm [9]. Source: https://www.dartmouth.edu/~humananatomy/figures/chapter_8/8-13_files/IMAGE001.JPG.

Regardless of whether management is operative or nonoperative, it is essential that patients with radial nerve injuries are enrolled in an active rehabilitation programme with involvement of a multidisciplinary team. Typically, the multidisciplinary team should include the surgeon, hand therapist, and physiotherapist. The use of passive range of movement exercises and dynamic splinting is vital in order to reduce joint stiffness and optimise hand and wrist function. Examples of the static and dynamic splints used in this case are shown in Figure 6.

## Figures and Tables

**Table 1:** 

Timepoint	Wrist extension (MRC grade)	Finger extension (MRC grade)	Radial nerve sensation
Initial injury	5/5	5/5	Slight paraesthesia
Immediately after Bohler-U cast	0/5	0/5	Absent sensation
1/52 post-op	0/5	0/5	Minimal sensation to light touch
6/52 post-op	1/5	3/5	Subjectively improving
3/12 post-op	3/5	3/5	Subjectively improving
8/12 post-op	5/5	5/5	Subjectively normal

## Additional Points

The importance of detailed documentation of neurovascular status, both at time of initial presentation and following any subsequent fracture manipulation, is highlighted by this case. Without a thorough clinical assessment and documentation of findings patient management may be compromised. In the presented case, the fracture configuration was suitable for conservative management. However, the specific intervention of fracture manipulation and Bohler-U cast application resulted in the patient developing a dense radial nerve palsy. In such circumstances, the clinician must assume that the reduction maneuver has resulted in the radial nerve becoming interposed in the fracture and surgical exploration is indicated. Though the clinical scenario in this case is very rare, failure to accurately determine the timing of onset of any neurological deterioration can detrimentally affect patient outcomes. The management of neurological injury sustained at time of initial injury and neurological injury sustained following fracture manipulation differs greatly. The basic principle of treating the patient, not just the X-ray, is reinforced.
